# Immunometabolism, extracellular vesicles and cardiac injury

**DOI:** 10.3389/fendo.2023.1331284

**Published:** 2024-01-08

**Authors:** Ana C. M. Omoto, Jussara M. do Carmo, Alexandre A. da Silva, John E. Hall, Alan J. Mouton

**Affiliations:** Department of Physiology and Biophysics, University of Mississippi Medical Center, Jackson, MS, United States

**Keywords:** myocardial infarction, microRNAs, metabolism, immune system, macrophages

## Abstract

Recent evidence from our lab and others suggests that metabolic reprogramming of immune cells drives changes in immune cell phenotypes along the inflammatory-to-reparative spectrum and plays a critical role in mediating the inflammatory responses to cardiac injury (e.g. hypertension, myocardial infarction). However, the factors that drive metabolic reprogramming in immune cells are not fully understood. Extracellular vesicles (EVs) are recognized for their ability to transfer cargo such as microRNAs from remote sites to influence cardiac remodeling. Furthermore, conditions such as obesity and metabolic syndrome, which are implicated in the majority of cardiovascular disease (CVD) cases, can skew production of EVs toward pro-inflammatory phenotypes. In this mini-review, we discuss the mechanisms by which EVs may influence immune cell metabolism during cardiac injury and factors associated with obesity and the metabolic syndrome that can disrupt normal EV function. We also discuss potential sources of cardio-protective and anti-inflammatory EVs, such as brown adipose tissue. Finally, we discuss implications for future therapeutics.

## 1 Role of immune cells and immunometabolism in cardiac injury and remodeling

Immune cells, including neutrophils, macrophages, and B and T lymphocytes, play a critical role in inflammation and repair after cardiac injury (1). While small resident populations of immune cells reside in the healthy heart, the majority of immune cells in the injured heart derive from extra-cardiac sources, primarily from the spleen (2). An acute inflammatory response is necessary to initiate the proper healing response to cardiac injury. Immune cells play a wide range of roles in the injured heart, including phagocytosis of necrotic cells and coordinating remodeling of the vasculature and extracellular matrix.

Recent studies have highlighted the importance of cardiac immunometabolism, the study of metabolic pathways that contribute to immune cell phenotypes (1, 3). The current paradigm, broadly speaking, is that inflammatory subsets of monocytes/macrophages or T cells (i.e. M1-like macrophages, Ly6C-high monocytes, Th1/Th17 cells) rely mainly on glycolysis, while anti-inflammatory/reparative subsets (M2-like macrophages, Ly6C-low/resident cardiac macrophages, Tregs) rely more on mitochondrial oxidative phosphorylation (OXPHOS). Glycolysis can allow cells to survive in a hypoxic environment, such as the ischemic heart, and permits activation of the pentose phosphate pathway (4). Macrophages and T cells can also program their metabolism to glycolysis even when oxygen is present, a phenomenon known as the Warburg effect. This glycolytic switch is mediated by activation of hypoxia-inducible factor-1 alpha (HIF-1α), which is activated by hypoxia or non-hypoxic signals such as toll-like receptor-4 (TLR4). Conversely, the mitochondrial tricarboxylic acid (TCA) cycle and OXPHOS promote pathways in immune cells that favor anti-inflammatory polarization (1).

## 2 Role of extracellular vesicles in cardiovascular health and disease

**Figure 1:**
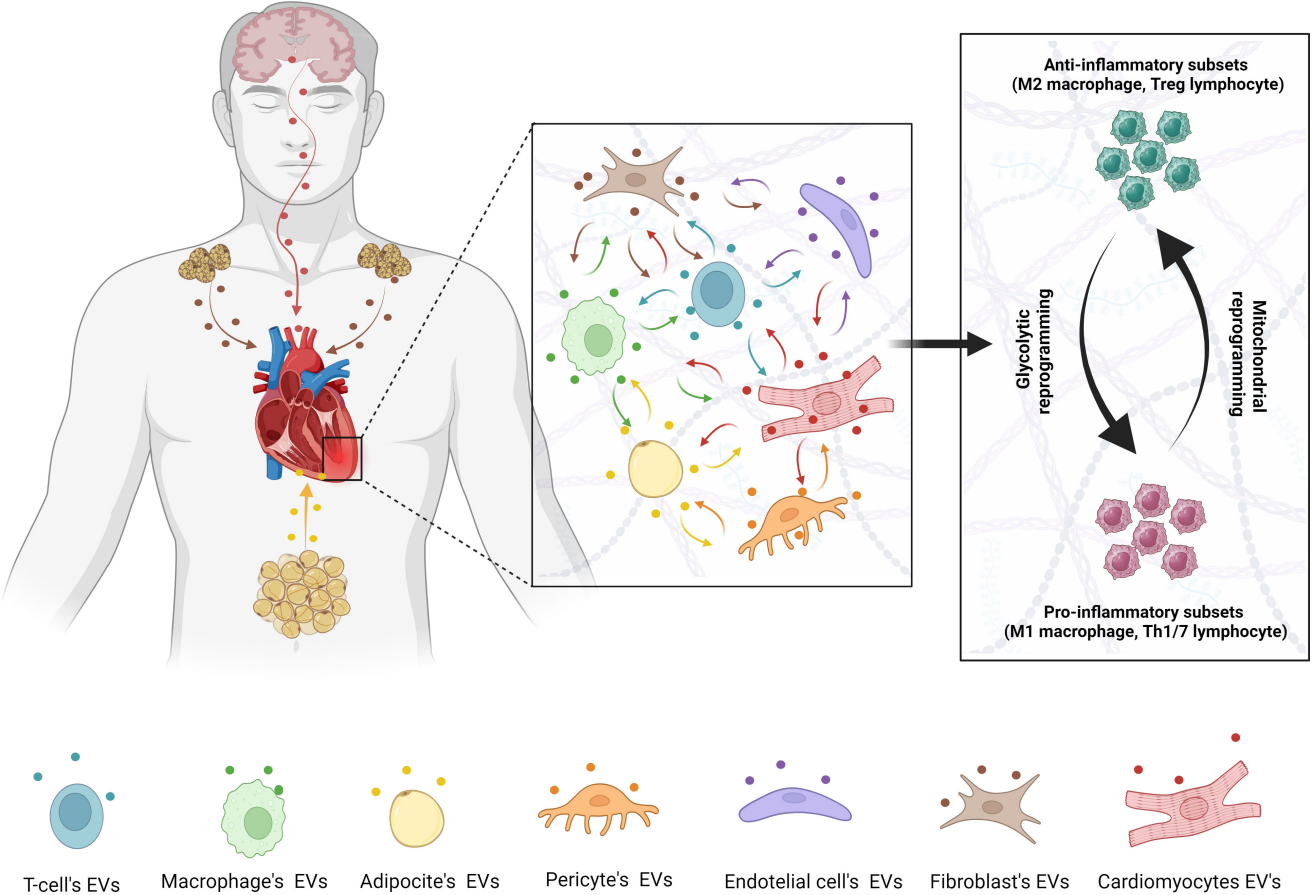
Metabolic regulation of inflammatory pathways during cardiac injury. Metabolic switching between glycolysis and mitochondrial oxidative phosphorylation is regulated by EVs from different cells population and distant organs.

While immunometabolism appears to be important for immune cell responses to cardiac injury, little is known of the upstream signaling mechanisms that drive metabolic reprogramming. One potential unexplored mechanism is through extracellular vesicles (EVs), which are released as membrane bound vesicles to mediate inter-organ communication in many physiological and pathological processes (**Figure 1**) (5). Due to their small size (30 nm – 4 μm) and the fact that EVs contain cargo that can behave as endocrine signaling molecules, there is much interest in EVs as therapeutic agents (5). EVs can also mediate communication between different cell populations of the cardiovascular system controlling normal tissue function or propagating injury signals during cardiovascular diseases. In contrast, certain EVs have also been identified to mediate cardioprotective mechanisms and have been proposed as promising drug delivery systems due to their low immunogenicity, toxicity and strong ability to cross cell membranes (6).

EVs can carry many different types of bioactive molecules within their membrane, including proteins, lipids, DNA, microRNAs (miRNAs), long non-coding RNAs (lncRNAs), circulating RNAs (cirRNAs) and mitochondria (7). The phenotype of the parent cell and its environment dictates EV’s cargo and how it will influence the target cell and inter-tissue crosstalk. EVs are generated by 3 different process: 1) fusion of multivesicular bodies with the cell membrane of the parent cell (exosomes, 30 – 100 nm), 2) outward budding of the parent cell membrane (microvesicles, 200 nm – 1 μm) and 3) membrane decomposition of apoptotic cells (apoptotic bodies, 1 – 4 μm) (8). Regardless of the biogenesis pathway, EVs contain in their membrane ligands inherited from their donor cells that could, theoretically, be used to identify the source of circulating EVs. However, studies are needed to elucidate specific molecular makers/ligands that each cell type shares with their EVs.

The content and mode of EVs interaction and/or entry into the target cell determine their functional effects. For example, EVs can trigger signaling pathways through interaction with receptors in recipient cells without being internalized (9). Also, EVs can directly enter into the cytoplasm of the recipient cell through endocytosis, phagocytosis or membrane fusion, subsequently releasing their contents into the cytoplasm (9). However, the most efficient way to transfer EVs content to the target cell in the context of immunometabolism modulation remains elusive.

Studies investigating EVs as potential biomarkers for cardiac diseases found that their cargo can be used as a signature for specific diseases. For example, circulating levels of EV-enclosed miR-126 and miR-199a can predict the occurrence of cardiovascular events in patients with stable coronary artery disease (10). Thus, EVs hold great potential to monitor and treat cardiovascular diseases but there are still many unanswered questions related to the regulatory mechanisms of EV biogenesis, selective sorting of cargo, and cell-specific EV uptake.

### 2.1 Inter-cellular communication during cardiac injury

The cardiovascular system is formed by a complex network of different cell types including cardiomyocytes, fibroblasts, endothelial cells, pericytes, adipocytes and immune cells. EVs play an important role in their interactions to maintain cardiac structure and function in healthy conditions. However, these cells also release EVs to propagate injury signals during disease. Evidence for a potential role of EVs during cardiac injury is suggested by increased circulating levels of EVs in patients with myocardial infarction (MI) as well as murine and porcine models of MI (11–13). EVs also accumulate in the heart early after MI where they may play a crucial role in regulating inflammation during the first 24 hr after MI (14).

EVs derived from necrotic cardiomyocytes are engulfed by phagocytic monocytes to promote release of cytokines such as IL-6, CCL2, and CCL7 (14). Endothelial cells are also potent sources of EVs after MI, as evidenced by cell surface expression of endothelial markers such as CD31, ICAM-1, PCAM-1, and P and E selectins (13). Endothelial-derived EVs are also engulfed by infiltrating monocytes, in which they promote inflammatory and migratory immune cell responses. Injecting naïve mice with these MI-derived EVs promoted monocyte mobilization from the spleen. For example, endothelial-EVs from diabetic mice impair angiogenesis and re-vascularization after MI and skeletal muscle ischemia (15, 16).

EV-mediated communication between immune cells and fibroblasts is proposed to be involved in cardiac fibrosis. Macrophages exposed to high glucose, mimicking a diabetic environment, produce EVs containing Human antigen R that stimulates fibroblasts to increase collagen production (17), thus enhancing cardiac fibrosis. Also, injection of CD4+ T cell-derived EVs carrying miR-142-3p exacerbates the effects of MI on cardiac function and infarct size expansion in mice (18). On the other hand, cardiac fibroblasts also produce EVs that propagate hypertrophic signals to cardiomyocytes during cardiac pressure overload (19). In addition, EVs produced by activated fibroblasts (myofibroblasts) may mediate endothelial dysfunction during cardiac fibrosis via miR-200a-3q (20).

Pericytes are another cardiac cell population that release EVs during cardiac injury, especially in ischemic diseases. These EVs are involved in cardiac fibroblast and macrophage proliferation (21). Also, crosstalk between pericytes and endothelial cells during inflammation in the heart is mediated by EVs in a bi-directional manner (22).

Epicardial adipose is another source of EVs that acts locally on the myocardium, atria and coronary arteries. Recently, these EVs were identified with proinflammatory, profibrotic and proarrhythmic properties that contribute to the development of atrial myopathy and atrial fibrillation (23). Altogether, these studies support an important role of EVs in the pathophysiology of cardiac diseases and demonstrate a strong interaction among different cell populations that is mediated by EVs and their cargos. How EVs sort their cargo in distinct disease models remains to be elucidated, although some studies suggest the participation of distinct RNA-biding sites and protein post-translational modifications including ubiquitination (6).

### 2.2 Inter-organ communication during cardiac injury and cardioprotection

Studies using transgenic animals expressing membrane-target fluorescent markers (mT/mG mice) (24) allowed in vivo tracing of EVs and showed that EVs produced in one organ can be detected in another organ during pathophysiologic processes. These findings suggest that EVs mediate not only inter-cellular but also inter-organ communications. For example, EVs produced by the heart of animals with congestive heart failure were detected in the brain, contributing to sympathetic excitation mediated by oxidative stress (25).

White adipose tissue (WAT) is proposed to be an important source of EVs with cardiac effects as demonstrated by studies showing that WAT from obese mice induces macrophage activation in a TLR4-dependent manner favoring cardiometabolic complications (26). Also, injection of lean mice with WAT-derived EVs, produced in response to obesity-associated stress, shifted tissue-resident macrophage toward a proinflammatory phenotype (26). In a diet-induced obese mouse model, WAT-derived EVs showed increased levels of miRNA-130b-3p, which exacerbated cardiac ischemia-reperfusion injury due to downregulation of adenosine monophosphate kinase (AMPK) (27).

Contrary to the detrimental effects of WAT-derived EVs in the heart, the brown adipose tissue (BAT) has been shown to be a potent source of EVs with potential cardioprotective and anti-inflammatory properties (28). Exercise activates release of small EVs from BAT enriched with cardioprotective miRNAs such as miR-125b-5p, miR-128-3p, and miR-30d-5p, which inhibit cardiac myocyte apoptosis following I/R injury via inhibition of the TNF receptor associated 6/TNF receptor superfamily member 1B signaling pathway (28). BAT-EVs are also enriched with mitochondrial components that improve OXPHOS and restore cardiac function in obese mice (29). Similarly, Lin et al. demonstrated a cardioprotective effect mediated by EVs communication from BAT to myocytes and cardiac fibroblasts (30). However other studies proposed that during obesity BAT-EVs become enriched with inflammatory proteins as well as the glucose transporter GLUT1, which mediates glucose uptake and glycolytic reprogramming in macrophages (1, 31). Furthermore, during hypertension, BAT-EVs may transport inducible nitric oxide synthase (iNOS) to the heart, which could aggravate cardiac remodeling and hypertrophy (30). Thus, while BAT-EVs appear to be cardioprotective in the healthy state, they may become maladaptive in obese or hypertensive subjects.

Another extracardiac source of EVs with cardioprotective effects are mesenchymal stem cells (MSCs) which reside in the bone marrow (7, 32). MSC-derived EVs have attracted attention as a potential therapeutic for MI due to their anti-apoptotic effect on cardiomyocytes, and their anti-inflammatory/pro-reparative effect on immune cells such as macrophages (33). Thus, harnessing the beneficial MSC-EVs while inhibiting cardiac myocyte and endothelial EVs from the injured heart may be a promising therapeutic strategy.

Together these studies emphasize the importance of the cell environment in determining EV’s cargo and their function on the target cell. Further investigation is needed to understand how EVs are attracted by the target cell in the distant organ.

## 3 Potential interactions of extracellular vesicles and immune cell metabolism in the heart

EVs clearly play an important role in regulating inflammation during cardiac injury. However how they impact immunometabolic reprogramming is still not well understood. Recent studies suggest that EVs are involved in macrophage polarization in response to different metabolic environments, thus contributing to the pathological process of cardiovascular diseases. In this section we summarize how different EV’s cargo can contribute to this process (**Table 1**).

**Table 1:** Extracellular vesicle content and their biological effects in the immune system.

EV content		Biological Effects	References
**Non-coding RNAs**	miR-223	Inhibit lipopolysaccharide (LPS)-induced glycolysis and M1-like polarization	Dang C.P. & Leelahavanichkul A., 2020 (36).
	miR-33	Decrease fatty acid oxidation and enhance glycolysis, promoting macrophage M1 polarization	Gest J. et al., 2015 (37).
	miR-22	Reduce glycolysis/M1 polarization by targeting GLUT1	Kang Y.J., 2023 (38).
	miR-142	Promote glycolysis and exacerbate inflammation during myocarditis	Sun P. et al., 2020 (40).
	lncRNA TUG1	Promote M2 macrophage polarization by suppressing miR-9-5p on SIRT1	Wentao M. et al, 2021 (44).
	lncRNA-MRGPRF-6:1	Promote M1 macrophage polarization by activating TLR4, myeloid differentiation factor-8, and TLR4-MyD88-MAPK	Dan Hu. et al., 2022 (45).
	lnc-RNA-ASLNCS5088	Inhibit the effect of M2 macrophages in fibroblast activation	Chen J. et al., 2019 (46).
	circ-RNA-Rps5	Promote M2 macrophage polarization by targeting SIRT7 and miR-124-3p	Yang H. et al., 2022 (48).
	circUbe3a-enriched M2 macrophages	Promote cardiac fibroblasts proliferation, migration and phenotypic transformation enhancing fibrosis after MI	Wang Y. et al., 2021 (49).
**Proteins**	Membrane protein-1	Activate the NF-κB-glycolysis pathway	Fridman E.S., 2022 (51).
	GLUT1	Enhance glycolysis	Garcia N.A., 2016 (53); Yang M. et al., 2022 (54).
	Enzymes involved in fatty acid oxidation	Activate anti-inflammatory pathways	Clement E. et al, 2020 (55) Mouton A.J., 2020 (1).
**Lipids and metabolites**	Lipids	Activation of adipose tissue macrophages	Flaherty 3^rd^ S.E. et al., 2019.
	Ribose-5-phosphate and pentose phosphate pathways enzymes	Immune cell proliferation during MI	Mouton A.J.et al., 2023 (4) Harmati M. et al., 2021 (56).
**Mitochondria**	mtDAMPs	Trigger inflammation via the cGAS/STING/NF-κB pathway	Di Mambro et al., 2023 (62).
	Healthy mitochondria	Promote an M2-like phenotype	Sanz-Ros J. et al., 2023 (61) van der Vlist M. et al., 2022 (65).
	Damaged mitochondria	Enhance OXPHOS and M2-like polarization	Sanz-Ros J. et al., 2023 (61) Phinney D.G. et al., 2015 (64)

### 3.1 MiRNAs

Metabolic reprogramming requires finely tuned activation and simultaneous repression of multiple cell signaling pathways, metabolites, and genes that can be regulated by multiple miRNAs (34). Activation of HIF-1α is recognized as a critical mediator of glycolytic reprogramming, while AMPK is a major regulator of reprogramming to OXPHOS metabolism (34, 35). Several miRNAs that regulate inflammation also regulate HIF-1α and several glycolytic enzymes. For example, miR-223 targets HIF1a and PFK1 to inhibit lipopolysaccharide (LPS)-induced glycolysis and M1-like polarization in vitro and in mice with sepsis (36). Alternatively, miR-33 targets AMPK and carnitine palmitoyltransferase 1a (CPT1a) to decrease fatty acid oxidation and enhance glycolysis, promoting macrophage M1 polarization (37).

Some miRNAs more directly target immunometabolic pathways. For example, miR-22 is induced by TLR signaling, and reduces glycolysis/M1 polarization by targeting GLUT1 (38). Other miRNAs can target other glycolytic genes, such as hexokinase-2, phosphoglucose isomerase, and enolase (39). In CD4+ T cells, miR-142 regulates the MBD2-MYC axis to promote glycolysis and exacerbate inflammation during myocarditis (40). Multiple miRNAs also regulate lactate dehydrogenase (LDHA) in CD4+ T cells, thus favoring their reliance on OXPHOS and improving their ability to kill viruses such as HIV (35). Several miRNAs have also been discovered to regulate mitochondrial function and metabolic pathways, termed the mitomiRs (41). Collectively, miRNAs that inhibit glycolysis appear to decrease inflammation, while those that inhibit fatty acid oxidation/OXPHOS promote inflammation.

### 3.2 LncRNAs and circRNAs

The role of EVs enriched with long noncoding (lnc) RNA and circular (circ) RNA on macrophages phenotypic changes is beginning to be elucidated. However, the interaction of these subtypes of non-coding RNAs (ncRNAs) with metabolic pathways that control immune functions is not well established.

LncRNAs are the longest type of ncRNA that participate in gene transcription, translation and post-translational modification (42). Recent studies have shown that lncRNA can act as a sponge for miRNA and alter specific signaling pathways involved in macrophage polarization (43). For example, EV’s resealed by endothelial progenitor cells promote M2 macrophage polarization by suppressing miR-9-5p on SIRT1 through the transfer on lncRNA taurine upregulated gene 1 (TUG1), an important lncRNA that is downregulated during sepsis (44). In patients with coronary atherosclerosis, EVs carrying lncRNA-MRGPRF-6:1 promote M1 macrophage polarization by activating TLR4, myeloid differentiation factor-8, and mitogen-activated protein kinase (TLR4-MyD88-MAPK) (45). Also, blockade of lnc-RNA-ASLNCS5088-enriched EVs dampens the effect of M2 macrophages in fibroblast activation (46).

CircRNAs are evolutionary conserved, stable and endogenous ncRNAs with important biological effects on protein activity regulation, epigenetic modulation, and transcription and post-transcriptional events (47). Yang et al. demonstrated that EVs derived from hypoxic-pretreated adipose stem cell enclose circ-RNA-Rps5 that promotes M2 macrophage polarization by targeting SIRT7 and miR-124-3p during acute ischemic stroke (48). Conversely, Wang et al. showed that M2 macrophage-derived EVs carrying circUbe3a promote cardiac fibroblasts proliferation, migration and phenotypic transformation enhancing fibrosis after acute myocardial infarction (49). Hence, these studies emphasize the importance of additional research on new lncRNAs and circRNAs enclosed into EVs and their role on cardiovascular diseases.

### 3.3 Proteins

Recent proteomics analyses of various EVs with resolution down to the single EV level have transformed our knowledge of EV-protein transport (50). Multiple studies suggest that EV transport of metabolic enzymes may influence immunometabolism. EVs from cancer cells can transport signaling proteins such as latent membrane protein-1 to cancer-associated fibroblasts, which activate the NF-κB-glycolysis pathway (51). Glycolytic enzymes are often enriched in EVs, regardless of origin (51, 52). EVs use glycolysis as an ATP source while also delivering glycolytic enzymes to recipient cells. Cardiomyocytes secrete EVs that can deliver the glucose transporter GLUT1, the major glucose transporter in macrophages (1), to neighboring cells to enhance glycolysis (53). Monocyte-derived EVs can also transport GLUT1 in addition to pro-inflammatory cargo, thus supporting inflammation through activation of glycolytic pathways (54). Conversely, EVs can also transport enzymes involved in fatty acid oxidation (55), which promotes anti-inflammatory pathways in macrophages and T cells (1).

### 3.4 Metabolites and lipids

EVs also carry other cargo that could influence immunometabolic phenotypes, including metabolites such as sugars, lipids, and amino acids (56). While most studies have focused on the protein/nucleic acid composition of EVs until recently, several studies have begun to focus on the EV lipid and small metabolite composition (56–58).

Obesity and metabolic disorders often lead to excess fat deposition in several organs including the heart. Within the adipose tissue, lipids regulate immune cells such as adipose tissue macrophages (ATM). Accumulation of lipids inside ATM activates a program of lysosomal catabolism, a process associated with systemic metabolic complications such as insulin resistance (59). Aiming to study how lipids are transferred from adipocytes to ATM, Flaherty III et al. conducted a series of experiments showing that adipocytes release EVs enriched with lipids, specifically triacylglycerol and monoacylglycerides, that were taken up by ATM (59), which can lead to metabolic disorders and development of cardiac diseases.

EVs enriched with ribose-5-phosphate and pentose phosphate pathways enzymes, may support immune cell proliferation during MI (4, 56). Indeed, many other small sugar, amino acid, and nucleotide metabolites have been discovered in EVs, depending on the source, size, and cardiovascular disease context (60).

### 3.5 Mitochondria

EVs can also transport mitochondrial enzymes, mitochondrial DNA, parts of the electron transport chain, mitochondrial fragments, and whole mitochondria (61). The role of these “mitoEVs” carry many implications for inflammatory pathways, which are tightly regulated by mitochondrial function (62). For example, mitochondrial damage-associated molecular patterns (mtDAMPs), which are released from damaged heart muscle, can trigger inflammation via the cGAS/STING/NF-κB pathway (62). Delivery of whole mitochondria to macrophages via EVs also plays a critical role in macrophage polarization (63). Damaged mitochondria can be packaged into EVs and sent to neighboring cells for repair (61). For example, MSCs undergoing mitophagy package damaged mitochondria into EVs, which are delivered to neighboring macrophages that complete the process of mitophagy, resulting in enhanced OXPHOS and M2-like polarization (64). Furthermore, healthy mitochondria from apoptotic cells can be “recycled” after being taken up by macrophages, in which they promote an M2-like phenotype (61). Conversely, macrophages can “donate” their mitochondria to injured or dysfunctional tissue, such as neurons (65). Thus, the role of mitoEVs in inflammation depends on the size, source, and contents of the EVs.

## 4 Gaps in knowledge and future implications

While several studies have implicated roles for EVs and their contents in regulating immunometabolism, the extent to which this occurs during cardiac injury remains unclear. Inflammation is a promising target for preventing adverse HF outcomes (66), although optimizing the therapeutic window remains a challenge. Targeting metabolism in immune cells is a promising therapeutic option due to availability of bioactive molecules that are well tolerated, such as NAD+ (67). Combining the potential delivery of “immunometabolic agents” with the specificity of EVs could provide a safe, effective method of delivering miRNAs, enzymes, functional mitochondria, or drugs to target immunometabolic pathways. However, several challenges still remain. There are multiple different types of EVs that may act on immune cells in the injured heart, based on size, cell surface expression, cargo, and source (33). Additionally, EVs carry a plethora of molecules with unknown effects, and changes in the donor cell environment can alter EVs cargo, thus complicating the predictability of EVs-based therapies (68). Furthermore, EVs can have a short plasma half-life imposing another challenge with regard the establishment of dose efficiency and off-target effects (68). Technical challenges also need to be overcome, including better loading of the therapeutic agents into EVs, targeting to specific cells within the same organ, and efficient delivery and uptake by target cells. Further understanding of these parameters during different types of cardiac injury, at different stages of disease, in different pre-clinical models (e.g. large animal models), and the impact of certain risk factors (e.g. obesity/diabetes) are still needed. As these fields continue to co-evolve and mature, it is likely that new therapeutic approaches for treating heart disease will emerge.

## Author contributions

AO: Writing – original draft, Writing – review & editing. JdC: Writing – original draft, Writing – review & editing. AdS: Writing – original draft, Writing – review & editing. JH: Writing – original draft, Writing – review & editing. AM: Writing – original draft, Writing – review & editing.
